# Cloud Prediction of Protein Structure and Function with PredictProtein for Debian

**DOI:** 10.1155/2013/398968

**Published:** 2013-07-18

**Authors:** László Kaján, Guy Yachdav, Esmeralda Vicedo, Martin Steinegger, Milot Mirdita, Christof Angermüller, Ariane Böhm, Simon Domke, Julia Ertl, Christian Mertes, Eva Reisinger, Cedric Staniewski, Burkhard Rost

**Affiliations:** ^1^TUM, Department of Informatics, Bioinformatics & Computational Biology-I12, Boltzmannstraß 3, 85748 Garching, Germany; ^2^Columbia University, Department of Biochemistry and Molecular Biophysics and New York Consortium on Membrane Protein Structure (NYCOMPS), 701 West 168th Street, New York, NY 10032, USA; ^3^Biosof LLC, 10th Floor, 138 West 25th Street, New York, NY 10001, USA; ^4^WZW-Weihenstephan, Alte Akademie 8, Freising, Germany; ^5^Institute for Advanced Study (TUM-IAS), Lichtenbergstraß 2a, 85748 Garching, Germany

## Abstract

We report the release of PredictProtein for the Debian operating system and derivatives, such as Ubuntu, Bio-Linux, and Cloud BioLinux. The PredictProtein suite is available as a standard set of open source Debian packages. The release covers the most popular prediction methods from the Rost Lab, including methods for the prediction of secondary structure and solvent accessibility (profphd), nuclear localization signals (predictnls), and intrinsically disordered regions (norsnet). We also present two case studies that successfully utilize PredictProtein packages for high performance computing in the cloud: the first analyzes protein disorder for whole organisms, and the second analyzes the effect of all possible single sequence variants in protein coding regions of the human genome.

## 1. Background

Bioinformatics is embracing cloud computing. Recent months have seen the publication of cloud sequence analysis platforms, CloVR [1] and Galaxy Cloud [2], and the cloud version of Bio-Linux [3], Cloud BioLinux [4]. Cost analysis depicts cloud computing as an attractive and sustainable solution for computational biology and bioinformatics [5–8]. The rate of data generation of “next generation” sequencing (NGS) drives the efforts to turn to cloud computing as a solution to handling peak-time loads, without the need to maintain large clusters [9]. Cloud-enabled bioinformatics tools are now available in the context of high throughput sequencing and genomics [10].

**Figure 1:**
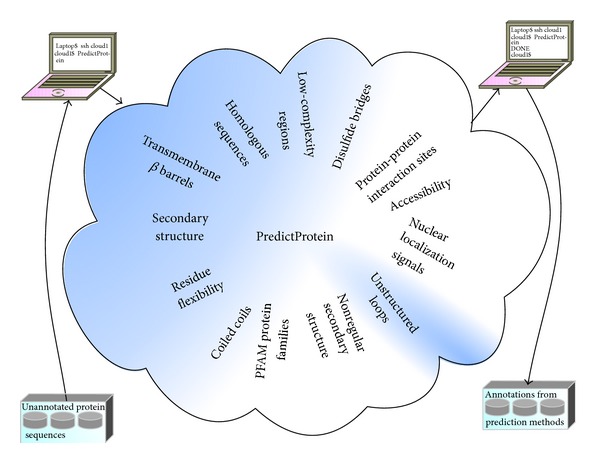
Protein annotation by PredictProtein. PredictProtein annotates input sequences with the features shown.

The Rost Lab provides protein structure and function prediction tools for cloud computing in the PredictProtein suite [11]. PredictProtein began as an Internet server for sequence analysis and the prediction of aspects of protein structure and function in 1992 [12]. Queried with a protein sequence, PredictProtein returns secondary structure and accessibility predictions, predictions of unstructured loops, nuclear localization signals, protein-protein interaction sites, disulfide bonds, regions lacking regular secondary structure, protein family hits, low-complexity regions, bacterial transmembrane beta barrels, coiled-coil regions, protein residue flexibility, and homologous sequences (Figure 1).

Cloud computing is commonly realized on machine instances that run on virtual hardware providing “infrastructure as a service” (IaaS) [13, 14]. This type of cloud computing instantiates compute nodes from machine images. Machine images usually contain an operating system with software tools. For example, one could request the instantiation of 10 worker nodes of PredictProtein on Debian operating system at the Amazon EC2 IaaS offering.

The PredictProtein cloud solution builds upon the open source operating system Debian [15] and provides its functionality as a set of free [16] software packages. Bio-Linux is an operating system for bioinformatics and computational biology. The latest Bio-Linux release 7 provides more than 500 bioinformatics programs on an Ubuntu Linux base [17]. Ubuntu is a “derivative” operating system [18] based on Debian, with its own additions. Cloud BioLinux is a comprehensive cloud solution that is derived from Bio-Linux and Ubuntu. Debian derivatives can easily share packages between each other. For example, Debian packages are automatically incorporated in Ubuntu [19] and are also usable in Cloud BioLinux (the procedure is described in [4]).

## 2. Implementation

The PredictProtein suite is implemented as a set of free packages released at http://debian.org/. Software packaging conformed with the Policy Manual [20], and following the recommendations of the Developer's Reference [21].

## 3. Results and Discussion

High-throughput experiments generate vast amounts of data at an ever-increasing rate; the pace of creating reliable annotations needed to use that data increases much slower. One of the major challenges for computational tools is to narrow the resulting increase in the protein annotation gap [22]. Of the over 35 m (million) sequences in the UniProt Knowledgebase 2013_05 [23], only about 500 k (500 thousand) have explicit experimental annotations in Swiss-Prot [24]. Computational prediction methods, such as those included in PredictProtein, can annotate important features for the remainder and enable us to draw scientific insights. Unfortunately, the task is often intractable for any single desktop computer within reasonable time. Fortunately, cloud computing is now at hand. On-demand servers in the cloud promise to fit computing power to most tasks economically, and without a fair portion of the usual worries of system management: hardware purchasing, recruiting a system manager, high availability issues, and so forth ([13] and the references therein). One problem remains: how to get the often adhoc analysis toolset from the desktop environment into the cloud? Directly addressing this problem, here we report the first Debian package release of the protein feature prediction toolset “PredictProtein,” developed at the Rost Lab.

**Figure 2:**
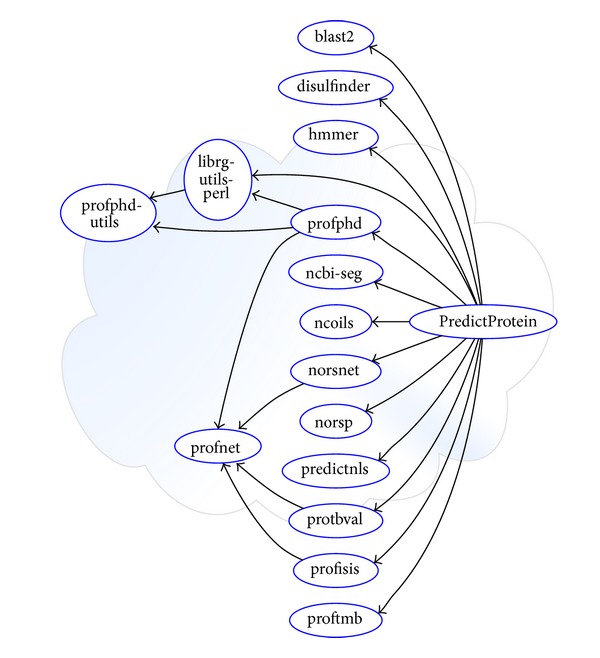
Package dependencies for PredictProtein. Arrows represent “depends on” relationships. Only significant dependencies are shown for clarity. Convenience copies of “profnet” for “profphd,” “norsnet,” “profbval,” and “profisis” have been merged to a single “profnet” package. Similar merging was done for all code convenience copies.

The publication of scientific results has, overall, changed surprisingly little since the Internet exists [25]. Research code is regularly distributed as a “zip” file of the development directory. Often, the only “documentation” distributed along with the code is the published paper accompanied by some “README” file. Software distributed this way often fails outside the laboratory without expert attention. In order to address this issue in the PredictProtein suite, we decided to apply the community and time-tested packaging and release requirements of Debian to PredictProtein components. We have traced all dependencies, eliminated convenience copies, carefully documented each of our prediction methods, and made them go through the thorough review process every Debian package receives. This converted PredictProtein from an adhoc implementation to a reusable software component (Figure 2).

Our packages facilitate the generation of purpose-built machine images for cloud computing. As an example, we distribute a slim PredictProtein machine image (PPMI) through the PredictProtein website [26]. This image contains a minimal installation of Debian with the command line version of PredictProtein. Databases are provided as a separate disk image. The PPMI is bootable on server instances in cloud infrastructure services, or on locally installed virtualization software. The latter allows for a cross-platform solution to use PredictProtein. Apart from virtualization, “chroot” environments present an option to run the software on Linux distributions where Debian packages are not readily usable. After booting the machine image, a friendly message at the login prompt offers usage tips and directions to documentation. A “Getting Started with PredictProtein” guide is available online [27]. The PPMI and the data image are updated regularly and are freely available at http://predictprotein.org/. For a comprehensive bioinformatics and computational biology computing environment, we recommend using PredictProtein with Bio-Linux [3] or Cloud BioLinux [4], where PredictProtein is either preinstalled or is easily installable from package repositories. We plan to release the web-based graphical interface of PredictProtein for these platforms in the near future.

The PredictProtein suite has attracted respectable popularity both online and offline. PredictProtein has been operating continuously since 1992, that is, the dawn of the Internet. Today, over 100,000 online users are registered; over 500 users access the PredictProtein web page every day and 12,000 unique users apply the service every month. Our Media Wiki page presenting an overview of the Rost Lab software packages has been accessed nearly 60,000 times since its launch 36 months ago. Adoption of the PredictProtein packages by the community has also been remarkable. Over 200 packages of the PredictProtein suite are installed from the Debian repository alone, while these and other installations have performed over 57 million protein feature predictions over the past year, not counting our own usage. Out of this, ~30 million were secondary structure and accessibility predictions from the “profphd” method [28].

## 4. PredictProtein Packages

The following protein feature prediction methods—components of PredictProtein—are available (feature—“package name”): secondary structure, accessibility, and transmembrane helices—“profphd” [29–31]; unstructured loops—“norsnet” [32]; nuclear localization signals—“predictnls” [33]; protein-protein interaction sites—“profisis” [34]; disulfide bridges—“disulfinder” [35]; nonregular secondary structure—“norsp” [36]; PFAM hits—“hmmer” [37, 38]; local complexity—“ncbi-seg” [39]; bacterial transmembrane beta barrels—“proftmb” [40]; coiled-coils—“ncoils” [41]; protein residue flexibility—“profbval” [42]; sequence homologies—“blast2” [43]; protein feature prediction suite—“predictprotein” [11].

These tools are available under a free license through Debian and are automatically incorporated into other Linux distributions such as Ubuntu. An overview of the packages offered for bioinformatics and cloud computing, complete with literature references, is available at Debian Med [44]. PredictProtein is listed in the Biology task.

## 5. Case Study 1: Protein Disorder in Completely Sequenced Organisms

The goal of this study is to collect evidence for three hypotheses on protein disorder: (1) it is more useful to picture disorder as a distinct phenomenon than as an extreme example of protein flexibility; (2) there are many very different flavors of protein disorder, but it is advantageous to recognize just two main types, namely, *well structured* and *disordered*; (3) nature uses protein disorder as a tool to adapt to different environments [45]. We predicted protein disorder both on an in-house compute grid and on a compute grid manually setup in the OpenNebula [46] cloud service provided by the CSC Finland [47]. Data and tool (the PPMI) images for grid nodes in the cloud were downloaded from http://predictprotein.org/. The PPMI image was extended with a grid client, and a separate machine instance was used as grid master. PredictProtein for the local grid was installed from the main Debian repository. Required databases (28 GB) were included on a data disk image for cloud machine instances. Input to PredictProtein jobs consisted of protein sequences (in total less than 1 GB). Grid job submissions to the local and the cloud grid were manually adjusted according to available resources. Over 9 million disorder predictions were made over the course of the past few years. 

## 6. Case Study 2: Comprehensive In Silico Mutagenesis of Human Proteome

This project aims at providing information about the functional effect of every possible point mutation in all human proteins, that is, for the replacement of 19∗N amino acids for a protein with N residues. Overall, this generated 300 million human sequence variants (point mutants). The method SNAP [48] predicted the effect of each variant, that is, each “nonsynonymous single nucleotide polymorphisms” (nsSNPs) upon protein function. These predictions are useful for several reasons. First, the study of all possible mutations in human will provide the background against which we can assess the effect of mutations that are actually observed between people. This is crucial for both the advance toward personalized medicine and health and the understanding of human diversity and variation. Second, our computation provides quick “look-up” answers available for all the important variants that are observed and implied in important phenotypes. The only way to cover those lookups is by precomputing all the possible changes. SNAP can take advantage of PredictProtein results for faster processing. With the PredictProtein packages presented here, a solution was built in the form of a public Amazon Machine Image (AMI, ami-3f5f8156) that allows running PredictProtein on the Amazon Elastic Compute Cloud (EC2). We extended an Ubuntu-based StarCluster [49] AMI with PredictProtein and its required databases (28 GB). Because every protein can be computed independently, we formed a grid job out of each protein and used the Grid Engine (GE) to distribute work on the machine instances. We used StarCluster to automate grid setup on the EC2. Because a lot of CPU power was needed, the “Cluster Compute Eight Extra Large Instance” was chosen. This instance type is especially crafted for big data with a lot of CPU power. One instance has 60.5 GB memory, 88 EC2 Compute Units (2x Intel Xeon E5-2670, eight-core-architecture “Sandy Bridge”), and 3370 GB instance storage. The sequence variants were analyzed based on the human reference proteome from the National Center for Biotechnology Information (build 37.3, proteins, 21MB). We processed 29,036 sequences with 16,618,608 residues. This amounted to predicting the functional effect of 315,753,552 individual amino acid changes.

## 7. Conclusion

The open source release of the PredictProtein protein structure and function prediction suite from the Rost Lab is now available for Debian and derivative operating systems, such as Ubuntu, Bio-Linux, and Cloud BioLinux. The software, due to its standard packaging, is readily deployable in the cloud. Successfully addressing the challenges of cloud computing brings PredictProtein—developed over almost two decades—into the present and the future. In accordance with the Rost Lab open policy [50], and supported by anonymous statistics, PredictProtein is now shared with a wide range of users. We encourage the bioinformatics community to take advantage of our open source software, itself a result of the collaboration of the wider open source software community.
